# Solid State Fermentation of a Raw Starch Digesting Alkaline Alpha-Amylase from *Bacillus licheniformis* RT7PE1 and Its Characteristics

**DOI:** 10.1155/2014/495384

**Published:** 2014-01-21

**Authors:** Romana Tabassum, Shazia Khaliq, Muhammad Ibrahim Rajoka, Foster Agblevor

**Affiliations:** ^1^National Institute for Biotechnology and Genetic Engineering (NIBGE), P.O. Box 577, Faisalabad, Pakistan; ^2^Department of Bioinformatics, G C University, Faisalabad, Pakistan; ^3^Biological Engineering Department, Utah State University, 4105 Old Main Hill, Logan, UT 84322-4105, USA

## Abstract

The thermodynamic and kinetic properties of solids state raw starch digesting alpha amylase from newly isolated *Bacillus licheniformis* RT7PE1 strain were studied. The kinetic values *Q*_*p*_, *Y*_*p*/*s*_, *Y*_*p*/*X*_, and *q*_*p*_ were proved to be best with 15% wheat bran. The molecular weight of purified enzyme was 112 kDa. The apparent *K*_*m*_ and *V*_max_ values for starch were 3.4 mg mL^−1^ and 19.5 IU mg^−1^ protein, respectively. The optimum temperature and pH for **α**-amylase were 55°C, 9.8. The half-life of enzyme at 95°C was 17h. The activation and denaturation activation energies were 45.2 and 41.2 kJ mol^−1^, respectively. Both enthalpies (Δ*H* ^∗^) and entropies of activation (Δ*S* ^∗^) for denaturation of **α**-amylase were lower than those reported for other thermostable **α**-amylases.

## 1. Introduction

Starch is an excellent carbon source in nature and major storage product of many economically important crops. A large-scale starch processing industry has emerged in the last century [1]. Five groups of enzymes play a key role in the hydrolysis of starch. These enzymes comprise about 30% of the world's enzyme production. Alpha amylases (endo-1,4-a-D-glucan glucanohydrolase, E.C.3.2.1.1) are extracellular endo enzymes that randomly cleave the 1,4-a linkage between adjacent glucose units in the linear amylose chain and ultimately generate glucose, maltose, and maltotriose units [2]. Alpha amylases derived from microbial sources have great success and replaced the chemical hydrolysis of starch in starch processing industries. Alpha amylases have a number of applications, including liquefaction of starch in the alcohol, brewing, and sugar industries and in the textile industry for desizing of fabrics [3]. They also have applications in laundry and detergents or as antisalting agents and baking such as bread making [3], since thermostability and alkaline characteristics are important features for industrial applications of amylase isolated from alkalophilic organisms [4].

The microbial *α*-amylases for industrial processes are derived mainly from *Bacillus subtilis, Bacillus amyloliquefaciens, *and* Bacillus licheniformis *asthey are capable of secreting amylases into the culture supernatant in submerged [5]. The microbial production of alpha-amylase is greatly influenced by the composition of the medium culture and environmental and growth kinetic parameters [5]. However, solid state fermentation provides an interesting alternative for concentrated enzyme production and less purification costs [6]. This work reports the solid state production, kinetic and thermodynamic properties of raw starch-digesting alkaline alpha-amylase from an indigenous culture of *B. licheniformis* RTPE1 which possessed potentially interesting properties from *α*-amylases already described.

## 2. Materials and Methods

### 2.1. Microorganism and Production Medium

Indigenous* Bacillus licheniformis* RTPE-1strain was maintained in NIBGE stock culture collection and 16S rRNA accession number EF644418 submitted at NCBI. The *Bacillus licheniformis* RTPE1 was maintained on maize starch (1% W/V) plates containing basal salts medium and 2.5% agar. The basal salt medium comprised of (g/L^−1^) K_2_HPO_4_ 1.0, (NH_4_)_2_ PO_4_ 1.0, MgCl_2_ 0.5, yeast extract 10.0, and maize starch 10. For liquid media preparation, all the media components were added and mixed one by one in one liter conical flask containing 200 mL distilled water. The pH of the medium was adjusted to 7.0 with 1N HCl/NaOH. The medium was autoclaved at 20 p.s.i, 120°C for 20 min.

### 2.2. Preparation of Inoculums

The basal salt medium containing maize starch (1% W/V) was dispensed in 50 mL quantity into 250 mL Erlenmeyer flasks. A single colony from fresh agar culture plate was transferred into the above medium and incubated at 37°C for 24 h on an orbital shaker (Gallenkamp, UK, 150 rpm).

### 2.3. Growth of Organism in Solid State Fermentation (SSF)

The SSF studies were carried out in one-litre Erlenmeyer flasks containing different concentrations of wheat bran, maize bran, and maize starch. Each carbon source was moistened with 25 mL basal salt solution. The flasks were autoclaved for 20 min and cooled. For time course study the flasks were inoculated with 5 mL of fresh inoculums (5 mg cell mass mL^−1^) and incubated at 37°C for 7 days. The incubator was humidified by sterile water. For the extraction of extracellular *α*-amylase from solid state fermentation experiments, after desired intervals (24, 48, 72, 96, 120, 144, and 168 h), the flasks were taken out in triplicate and the enzyme was extracted (three washings) with 100 mL of 10 mol phosphate buffer (pH 7.0) after vigorous shaking on an orbital shaker (150 rpm, 4°C). The extracted enzyme was centrifuged (at 15,000 ×g for 10 min at 4°C) to get clear supernatant for enzyme assay. All the experiments were run in three replicates.

### 2.4. Assay for Alpha-Amylase Activity

The activity of *α*-amylase was measured on the basis of liberated reducing sugars [7] using 3,5-dinitrosalicylic acid (DNS) using starch as the substrate after 10 min incubation. One unit of *α*-amylase activity was defined as the amount of enzyme, which released 1 *μ*mol of maltose or glucose equivalent per min under the assay conditions. Glucose was measured using glucose oxidase kit.

### 2.5. Protein Determination

The protein in the enzyme preparation was quantified by the Bradford method [8] using bovine serum albumin as the standard.

### 2.6. Purification of *α*-Amylase

All purification steps were conducted at room temperature. Enzyme extract from above (1000 mL) was purified by a combination of ammonium sulphate precipitation, ion-exchange, and gel filtration column chromatography as described previously [9] with 20-fold increase in specific activity (138 to 2769 U (mg^−1^ protein) with 30% recovery).

### 2.7. pH Optima Studies

The effect of pH on the stability of *α*-amylase was determined by assay of *α*-amylase activity after incubating the enzyme in the reaction system of different pH values at 50°C for 1 h [10].

### 2.8. Effect of Substrate Concentration

*α*-Amylase was assayed in 100 mM phosphate buffer of pH 7, with variable amounts (0.02%–1%) of soluble starch solution. The data was plotted according to Line-Weaver Burk plot as described [11].

### 2.9. Effect of Activators and Inhibitors on *α*-Amylase Activity

The enzyme solutions were incubated for 10 min along with activators and inhibitors with the final concentration of 1 mM at 55°C and then assayed in the presence of metal ions for *α*-amylase activity. The various activators or inhibitors used were HgCl_2_, CaCl_2_, CuSO_4_, MgCl_2_, ZnCl_2_, FeSO_4_, CaCl_2_, MnCl_2_, SDS, and EDTA.

### 2.10. SDS-Denaturing-Renaturing Polyacrylamide Gel Electrophoresis (SDS-DR-PAGE)

Subunit molecular weight of **α**-amylase was determined by subjecting crude and purified *α*-amylase to 10% SDS-PAGE using Hoeffer small apparatus. After electrophoresis, the gel was incubated for 30 min in 100 mmol potassium phosphate buffer (pH 7.5) containing 20% isopropanol for the removal of SDS. After removing SDS, the part of the gel containing different molecular weight markers was stained over night with Comassie *R*-250. The gel was destained with a solution of methanol, acetic acid and water with a ratio of 9 : 2 : 9, respectively. The part of gel containing *α*-amylase was transferred to potassium iodide solution (Gram's iodine) for activity staining [11].

### 2.11. Half-Life

Purified *α*-amylase was redissolved in 100 mmol phosphate buffer (pH 7) and assayed for thermostability. Optimum temperature of *α*-amylase was determined in 100 mM potassium phosphate buffer (pH 7.0) at selected temperatures (50, 55 60, 65, 70, 75, 80, 85, 90, 95 and 100°C) of reaction system [11, 12]. *α*-Amylase was examined by incubating the enzyme in the above buffer, at certain temperatures (50–90°C) and the enzyme samples were taken after 15, 30, 60, 120, and 180 min of incubation for the assay of enzyme activity.

### 2.12. Thermodynamics of Enzyme

Arrhenius relationship was used to calculate the activation energy (*E*_*a*_) required by the enzyme to hydrolyze starch as described earlier [11]. Thermal inactivation of the enzyme was determined by incubating the enzyme solutions in above buffer at a particular temperature. Aliquots were withdrawn at different times, cooled on ice, and assayed for enzyme activity at 55°C as described above. This procedure was repeated at different temperatures ranging from 45 to 100°C. The data were fitted to first-order plots and analyzed. The first-order rate constants (*k*_*d*_) were determined by linear regression of ln (*V*) versus time of incubation (*t*). The thermodynamic data were calculated by rearranging the Eyring absolute rate equation to study the overall thermodynamic parameters in the range 45–95°C:

(1) ΔS∗Kd=T·kB/heR  ΔeH∗R·T,ln⁡(kdT)=
ln
(kBh)+ΔS∗R−ΔH∗R·1T,

where *k*_*d*_, *T*, *k*_*B*_, *h*, ΔS*, ΔH*, and *R* are specific reaction velocity, absolute temperature, Boltzmann constant, enthalpy of activation, and gas constant, respectively.

## 3. Results and Discussion

Various factors including particle size, inoculum density, moisture content, and enzyme extraction parameters were employed when wheat bran, maize starch, and maize bran were used. For comparison of data to make extrapolations, IUg-1 cells (*Y*_*p*/*x*_), IUg-1 substrate consumed (*Y*_*p*/*s*_), volumetric productivity's (*Q*_*p*_), and specific productivity (*q*_*p*_) of the enzyme have been presented in Tables 1(a) and 1(b). Wheat bran (15%) proved to be the best carbon source to support maximum values of all kinetic parameters related to product formation. Maximum values of *α*-amylase *Q*_*p*_ (1302 IU L^−1 h^), *Y*_*p*/*x*_ (1333 IU^g−1^ cells), *Y*_*p*/*s*_ (22588 IU^g−1^ substrate consumed), and *q*_*p*_ (357 IUg^−1^ cells h^−1^ specific) were also improved (Table 1(a)). Growth yield coefficient (*Y*_*x*/*s*_), volumetric rate of substrate utilization (*Q*_*s*_), specific rate of substrate utilization (*q*_*s*_), specific growth rate (*μ*), and volumetric productivity of extracellular protein (*Q*_*pe*_) were also increased with 15% wheat bran (Table 1(b)).

The suitability of a particular substrate in a SSF process for the production of bacterial amylases appeared to be governed by the physicochemical requirement of the microorganisms used. The universal suitability of wheat bran may be due to the fact that it contains sufficient nutrients and is able to remain loose even in moist conditions, thus providing a large surface area. When compared to earlier studies in SSF [13], enzyme production was enhanced up to several in SSF and a 2.4-fold improvement in specific productivity was noted compared with submerged fermentation.

**Figure 1:**
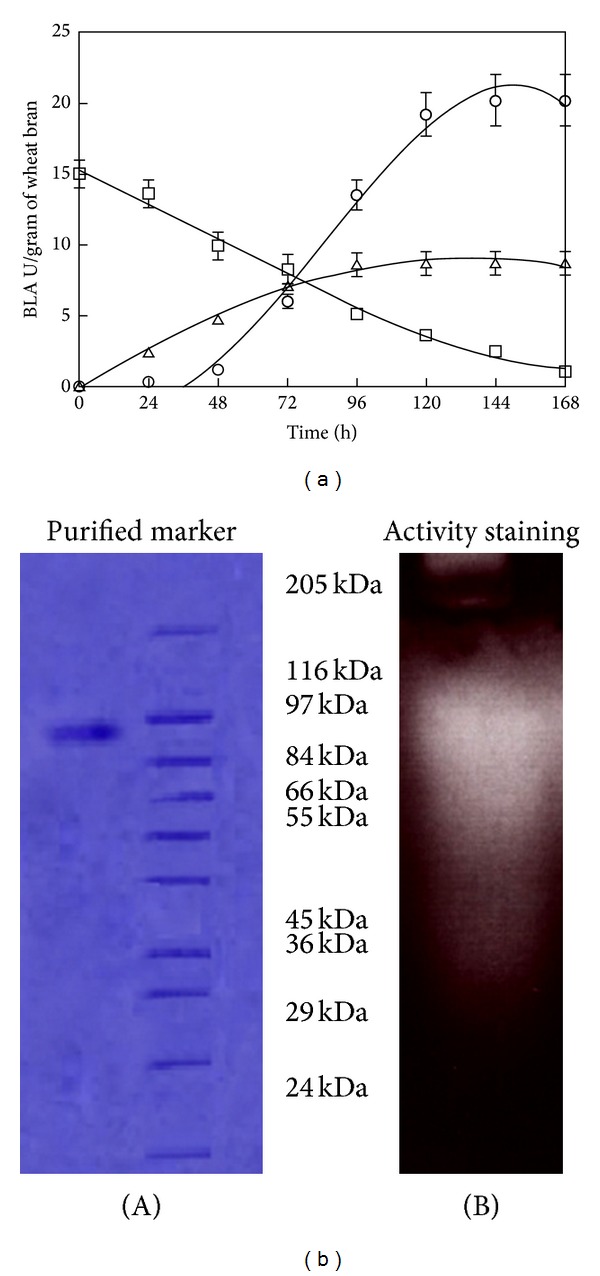
(a) Kinetics of alpha-amylase production in solid state fermentation using wheat bran. The initial pH of the medium was 7.0 and temperature 37°C. ○: alpha-amylase, Δ : *X* (cell mass), and □: *S* (substrate) present in the fermentation medium. Error bars show standard deviation among three replicates. (b) Purification profile of enzyme on SDS-PAGE: A, lane 1: purified enzyme; lane 2: molecular weight markers; B, lane 1: activity staining of purified enzyme.

The novel aspect of the study was that spore forming indigenous *Bacillus licheniformis* RTPE1 strain produced maximum amount of extracellular *α*-amylase when growth rate declined principally in the stationary phase (see representative Figure 1(a)), though maximum enzyme production was observed in the fermentation medium producing maximum amounts of biomass. In the earlier findings, mostly *α*-amylase production was growth associated and highest enzyme activities were obtained in the exponential phase and even in the early stage of growth phase [5]. Very few findings revealed that the production of *α*-amylase is nongrowth associated. Further enhancement in the catalytically active enzyme can be obtained by exploiting nitrogen sources and maintenance of proper aeration in the fermentation vessel.

### 3.1. SDS-Polyacrylamide Gel Electrophoresis of the Purified *α*-Amylase of Indigenous *Bacillus licheniformis* RTPE1 Strain

Purified *α*-amylase was subjected to SDS-PAGE as described in materials and methods. A single band of purified *α*-amylase was observed in lane 1 (Figure 1(b)A). It was found that the molecular weight of *α*-amylase was 112 kDa and enzyme was active *in situ* and showed zone of clearance even in crude samples (Figure 1(b)B). Purified *α*-amylase has been shown to be homogenous by SDS-PAGE. It has higher molecular weight than most of the *α*-amylases from *Bacillus sp* (40–140 kDa) [15].

### 3.2. Alkaline Nature of the Indigenous *Bacillus lichenformis* RT 7PE1 *α*-Amylase

**Figure 2:**
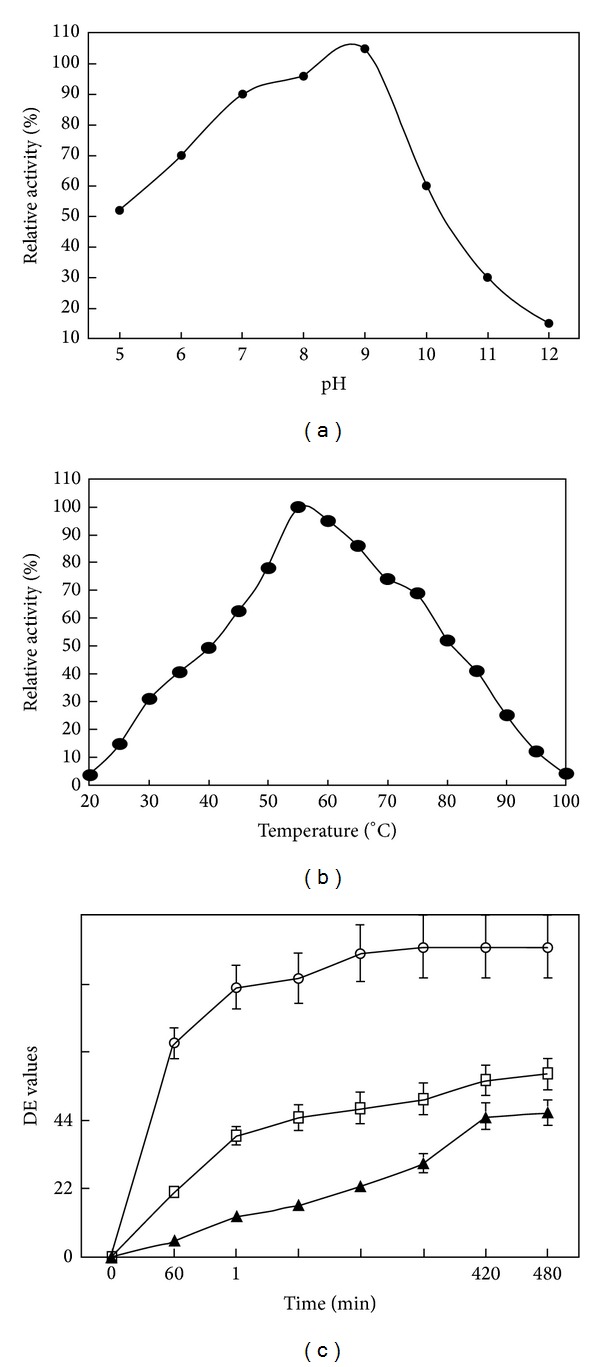
(a) Effect of pH on enzyme activity, (b) effect of temperature on enzyme activity, and (c) liquefaction of maize starch used at different concentrations: ▲ 10% maize starch, □ 20% maize starch, and ○ 30% maize starch. Enzyme was used 200 IU per g substrate at 55°C.

Studies of alkalophilic microorganisms had resulted in the discovery of extra-cellular enzymes that are characterized by maximum pH for activity and stability occurring on the alkaline side. Although various extra-cellular enzymes of alkalophilic microorganism have been described and only a few are alkaline amylases [16]. Maximum activity was observed at pH 9.0 and 60°C (Figures 2(a) and 2(b)), which is very suitable for industrial use. In previous study, the effect of temperature and pH on the activity of *α*-amylase, produced by *Bacillus* sp. KR-8104 in a solid state fermentation system, was optimized [17]. Comparing the results obtained from the optimization of crude *α*-amylase activity in solid state and submerged fermentation systems revealed some differences in pH and temperature optima for maximizing the crude enzyme activity.

### 3.3. Effect of Metal Ions and Effectors on *α*-Amylase Activity and Thermostability

The thermostability of purified enzyme was enhanced in the presence of 5 mM Ca^2+^ at 90°C. The enzyme activity was strongly inhibited by Hg^2+^, Pb^2+^, Zn^2+^, Cu^2+^, EDTA, and SDS (Table 2) [12].

But in the presence of 5 mM Ca^2+^ enzyme retained 97% of its activity even after incubation of 180 min at 90°C. In general high concentrations of both monovalent and divalent salts were inhibitory to the reaction. The inhibition depended upon the nature of the salt, either due to anionic strength effect or specific cation effect [10].

### 3.4. Liquefaction of Starch

Liquefaction of the 10% maize starch was done with purified *α*-amylase (200 IU g^−1^ starch) from indigenous *Bacillus licheniformis* RT7PE1 strain. The enzyme gave 100% dextrose equivalent (DE) values from 10% maize starch hydrolyzed completely after 360 min of incubation. Starch at 30% (w/v) was not completely hydrolyzed to glucose (Figure 2(c)). High performance liquid chromatographic studies proved that the starch hydrolysis major product was maltose and oligosaccharides. The production of sugars from starch sources is an industry that exists in its present form due to the application of industrial enzymology to solve process related problems. As the industry matures, the demand for more efficient enzymes leading to higher quality products and lower production costs for the starch processing will increase [18].

### 3.5. Effect of Starch Concentration on Catalytic Activity

**Figure 3:**
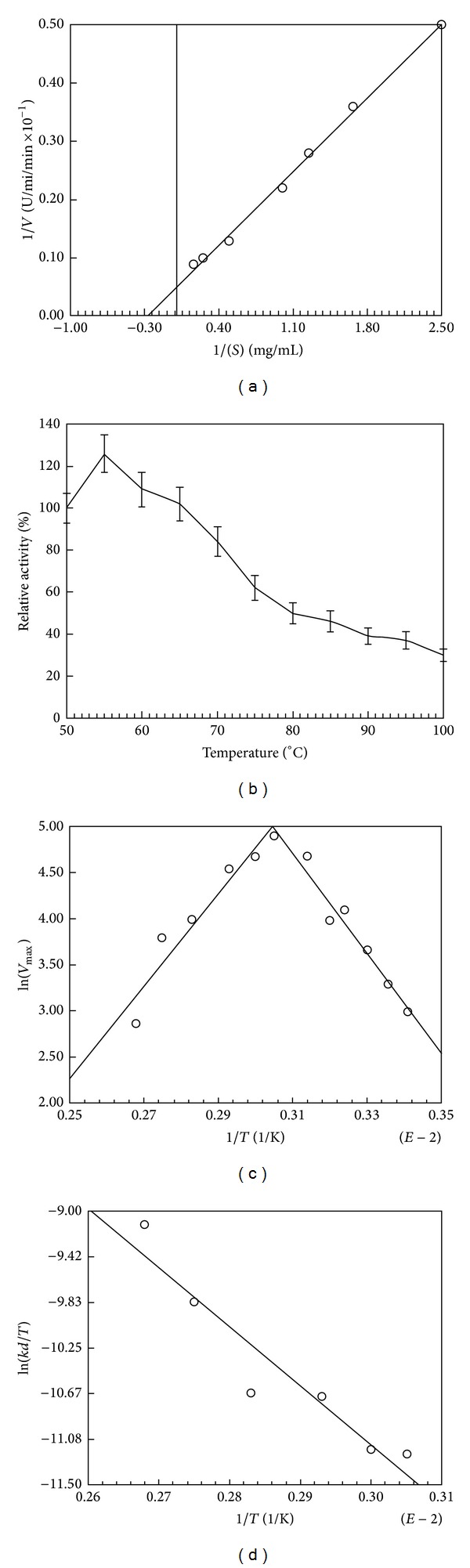
(a) Line weaver-Burk plot for calculation of *K*_*m*_ and *V*_max⁡_ of enzyme. (b) Determination of protein midpoint (*T*_*m*_) for denaturation. Relative activity at each temperature was calculated as described in Materials and Methods and plotted against temperatures. *T*_*m*_ is that temperature at which held of enzyme is defolded. (c) Arrhenius plots for calculation of activation energy. (d) Arrhenius plots for calculation of activation enthalpy and entropy of alpha-amylase inactivation.

The reaction was dependent on the amount of starch present in the reaction mixture. A Line-weaver Burk plot (Figure 3(a)) of the data reveals a *K*_*m*_ of 3.4 mg starch mL^−1^. The *V*_max⁡_ is 19.5 *μ*mol min^−1^. So the amylase exhibited Michaelis-Menten-type kinetics. The kinetic parameters of *α*-amylase activation *V*_max⁡_ and *K*_*m*_that were 263 *μ*mole mg^−1^ enzyme min^−1^ and 0.97 mg/mL, respectively, were reported from thermopile *Bacillus sphaericus* [14]. The *V*_max⁡_ and *K*_*m*_ values for soluble starch was found to be 4.11 mg/min and 3.076 mg, respectively, for *α*-amylase isolated from *B. amyloliquefaciens* previously reported by Gangadharan et al. [19].

### 3.6. Effect of Temperature on *α*-Amylase Activity and Stability

Stability of the enzyme at higher temperature up to 100°C was examined, and the enzyme was optimally active at 55–60°C (130 IU mL^−1^) (Figure 3(b)) and showed 14% relative enzyme activity at 100°C. The temperature-dependent properties of this enzyme show some similarity to *Bacillus licheniformisα*-amylase (BLA) produced by recombinant* E. coli *which was active over a temperature range of 50–80°C and had optimal activity at 60°C [20].

### 3.7. Half-Life and Activation Energy

The protein midpoint inactivation temperature (*T*_*m*_), activation energy, conformational stability at elevated temperatures, activation parameters for catalytic activity, transition state formation energy, stability of the native state ensemble are potential indices for thermostable biocatalysts. The *T*_*m*_ value was 89°C (Table 3(a)). For *Bacillus licheniformis* amylase (BLA) at pH 7.0, the *T*_*m*_ of 103°C was obtained [20].

Activation energy for catalysis of soluble starch was 45.2 kJ mol^−1^ (Table 3(a)). The activation energy for *B. licheniformis* CUMC305 enzyme was calculated as 5.1 × 105 J/mol. The investigated most thermostable *α*-amylases from *Bacillus licheniformis* enzymes exhibit activation energies (*E*_*a*_) between 208 and 364 kJmol^−1^ and pronounced differences in the corresponding unfolding rates. After the inflexion in temperature (Figure 3(c)), the enzyme becomes denatured and shows less activity towards conversion of substrate into product with decrease in activation energy (41.2 kJ mol^−1^) which infers thermostability of the test enzyme [21].

### 3.8. Kinetics of Starch Hydrolysis

The ratio *k*_cat_/*K*_*m*_ often referred to as the “specificity constant” is a useful index for comparing the relative rates of enzyme acting on alternative, competing substrates. In present studies, *k*_cat_ is 2395/min and the specificity constant *k*_cat_/*K*_*m*_ of this enzyme is 7068 (Table 3(a)). The reported *k*_cat_ for *α*-amylase from *B. amyloliquefaciens* is 2.26 × 10^3^ s^−1^ [22, 23]. This could be due to the ionized state of the carboxyl whose charges are positive ones and the substrate (full chair conformation of sugar residue) binds more strongly to the active site than the transition state of the substrate (sofa form of the positively charged oxo-carbonium ion). It was found that the entropy of denaturation (ΔS*) of alpha-amylase at 60°C was significantly decreased as compared with other enzymes indicating altered and more compact conformation for this enzyme. This happens due to the increase in the hydrophobicity around active site, as a result of local conformational change due to substrate (starch) binding and is a common phenomenon.

### 3.9. Thermodynamics of Starch Hydrolysis by Alpha-Amylase

The organism-derived enzyme requires less free energy (Δ*G*_*E*−T_*) to form the transition state than that required by other organisms (Table 3(a)). Similarly, enzyme releases the higher amount of transition state binding energy (Δ*G*_*E*−S_*) as compared with those of others (Table 3(a)), signifying that the high catalytic efficiency of alpha-amylase is due to the transition state stabilization. Accordingly, this enzyme showed highest enzyme-substrate destabilization (Δ*G*_*E*−S_*), whereas other enzymes showed least enzyme-substrate destabilization energy (Table 3(a)). The activation energy (*E*_*a*_) profiles of the enzyme show that this alpha-amylase has lower *E*_*a*_ values up to 90°C.

These studies provided insight into improvement in enzyme production and the process of stabilization by this strain. The culture had altered the values of both entropy and enthalpy of activation for irreversible inactivation of enzyme as observed for thermostabilized enzymes [24]. Actually thermal inactivation occurs in two steps as follows:

(2) $$\begin{array}{c} N⟷U⟶I, \end{array}$$

where *N* is the native, *U* is the unfolded enzyme which could be reversibly refolded upon cooling, and *I* is the inactivated enzyme formed after prolonged exposure to heat and therefore can not be recovered on cooling.

The thermal denaturation of enzymes is accompanied by the disruption of noncovalent linkages, including hydrophobic interactions, with concomitant increase in the enthalpy of activation [24]. The opening up of the enzyme structure is accompanied by an increase in the disorder, randomness or entropy of activation. The values of thermodynamic parameters were calculated from Figure 3(d). The enzyme had 44.8 kJ mol^−1^ and −155.6 J·mol^−1^ K^−1^ΔH* (enthalpy of deactivation) and ΔS* (entropy of deactivation) values respectively. The values of ΔS* and ΔH* of *β*-glucosidase from a thermophilic culture of *Aspergillus wentii* [24] were 125 kJ mol^−1^ and 65 J·mol^−1^ K^−1^, respectively. The values of BLA here are markedly lower, therefore, up to 95°C, the enzyme was reasonably thermostable. These values are also significantly lower than those reported on thermostable mutant of *Aspergillus awamori* glucoamylase [24]. When enthalpy and entropy values for inactivation were calculated at each temperature, ΔS* had again negative values (Table 3(b)). This suggested that there was negligible disorderness as was that of *β*-glucosidase from *A. wentii *or the transition state of *α*-amylase from* Bacillus licheniformis *was found to be more ordered as revealed by its negative ΔS* (−150 J mol^−1^ K^−1^) at high temperature of 80°C [25].

## 4. Conclusion

From the present study it is apparent that production of alpha amylase production by solid state fermentation from 15% wheat bran was higher as compared to 15% gram bran by newly isolated *Bacillus licheniformis *RT7PE1 strain. The wheat bran was best and cheap source for production of alpha amylase. The production of enzyme in submerged fermentation is expensive as compared to solid state fermentation. The contents of synthetic media are very expensive and these contents might be replaced with more economically agricultural waste material for the reduction of cost of the medium. The use of agricultural wastes makes solid state fermentation an attractive alternative method. There is on going interest in the isolation of new bacterial strains to produce amylases for new industrial applications, such as alkaline amylases for the detergent industry. This *α*-amylase can be used as a good model protein for investigation of the molecular basis of alkalophilicity of moderately thermostable alkaline enzymes.

## Figures and Tables

**Table:** (a)

Carbon source	*Q*_*p*_ IU L^−1^ h^−1^	Y_p/s_ IU g^−1^ substrate utilized	Y_p/X_ IU g^−1^ cells	q_p_ IU g^−1^ cells h^−1^ specific productivity
5% wheat bran	535 ± 25	1500 ± 10	11904 ± 25	357 ± 25
10% wheat bran	1262 ± 35	1550 ± 12	19375 ± 30	775 ± 28
15% wheat bran	1302 ± 29	1333 ± 15	22588 ± 35	1242 ± 30
10% maize starch	427 ± 41	1260 ± 14	10500 ± 26	315 ± 21
5% maize bran	867 ± 30	1420 ± 12	18933 ± 29	719 ± 12
10% maize bran	990 ± 45	1100 ± 13	16500 ± 31	858 ± 23
15% maize bran	851 ± 48	1450 ± 14	17682 ± 32	725 ± 25

Each value is a mean of three replicates. (±) stands for standard deviation among replicates. *Q_*p*_ = IU l^−1^ h^−1^, Y_p/s_ = IU g^−1^ substrate utilized, *Y*_*p*/*X*_ = IU g^−1^ cell, and *q*_*p*_ = specific productivity = IU g^−1^ cells h^−1^ and were determined as described previously [14].

**Table:** (b)

Carbon source	Biomass g	*Y*_*x*/*s*_	*Q*_*s*_	*q*_*s*_	*μ*	QP_*e*_
	g DW/Lh	g/g	g/L/h	g/gh	(h^−1^)	g/L/h
5% wheat bran	0.079	0.126	0.281	0.238	0.0300	0.153
10% wheat bran	0.084	0.080	0.351	0.560	0.040	0.271
15% wheat bran	0.095	0.062	0.554	0.887	0.055	0.300
10% maize starch	0.053	0.082	0.600	0.500	0.041	0.315
5% maize bran	0.052	0.120	0.285	0.250	0.030	0.145
10% maize bran	0.063	0.075	0.487	0.506	0.038	0.260
15% maize bran	0.0635	0.056	0.650	0.092	0.050	0.295

*Y*_*x*/*s*_: growth yield coefficient, *Q*_*s*_: volumetric rate of substrate utilization, *q*_*s*_: specific rate of substrate utilization, *μ*: specific growth rate, and QP_*e*_: volumetric productivity of extracellular protein. Each value is a mean of three replicates. (±) stands for standard deviation among replicates.

**Table 2:** Effect of metal ions (used at 5 mM) on enzyme activity.

Metal ions	% Relative activity
Control	100 ± 3
CaCI_2_·2H_2_O	115 ± 4
CoCl_2_·6H_2_O	23 ± 1
FeSO_4_·5H_2_O	20 ± 3
HgCI_2_	0 ± 0
KCI	67 ± 3
CuSO_4_	0 ± 0
MgSO_4_	97 ± 4
EDTA	30 ± 2
SDS	20 ± 2

Each value is a mean of three readings; ±: stands for standard deviation among replicates.

**Table:** (a)

Parameters	Values
*k*_cat_ (min^−1^)^a^	2395
*K*_*m*_ (%w/v)	0.34
*k*_cat_/*K*_*m*_	7043
*E*_*a*_ (kJ mol^−1^)^b^	45.2
*M*_*r*_ (kDa)	124
pH optimum	9.5
Temperature optima	55
*T*_*m*_ (Midpoint Temp)	89
ΔG* (kJ mol^−1^)^e^	−164.8
ΔH* (kJ mol^−1^)^f^	44.8
ΔS* (Jmol^−1^ K^−1^)^g^	−155.6
Δ*G*_E−T_* (kJ mol^−1^)^h^	−30.3
Δ*S*_E−S_*(kJ mol^−1^)^I^	−2.86

^a^Turnover number (*k*_cat_) = *V*_max⁡_/[*e*], where *e* is *α*-amylase concentration (0.00806 *μ* mol). NA: not available.

^b^Activation energies (*E*_*a*_) determined as described previously [11].

^e^ΔG* (activation free energy of *α*-amylase hydrolysis) = −  *RT*. ln (*k*_cat_·*h*)/(*K*_*B*_·*T*), where *h* is planck constant (6.63 × 10^−34^ Js), *K*_*B*_ is boltzman constant (1.38 × 10^−23^ JK^−1^), and *R* is gas constant (8.314 JK^−1^ mol^−1^).

^f^Δ*H*  (activation enthalpy of starch hydrolysis) *E*_*a*_ – *RT*.

^g^Δ*S*  (activation entropy of starch hydrolysis) = (ΔH* − ΔG*)/*T*.

^i^Δ*G*_*E*−*T*_  (free energy of transition state binding) = − *RT* ln *k*_cat_/*K*_*m*_.

^J^Δ*S*_*E*−*S*_  (free energy of substrate binding) = − *RT* ln *K*_*a*_, where *K*_*a*_ = 1/*K*_*m*_.

**Table:** (b)

*T*	*K*_*d*_	*t*_1/2_	ΔH*	ΔG*	ΔS*
	10^−3^ h^−1^	(h)	kJ mol^−1^	kJ mol^−1^	J mol^−1^ K^−1^
323	4.4	158	42.51	115.76	−226
328	4.7	147	42.47	117.53	−229
333	6.8	102	42.43	118.29	−230
343	7.8	89	42.35	121.59	−231
353	8.3	83	42.27	125.04	−234
363	18.0	38	42.18	126.33	−240
368	41.0	17	42.18	127.12	−231

^+^ *K*_*d*_ (first-order rate constant for inactivation) was calculated from relationship, *K*_*d*_·*t* = ln *V*, where *t* is time of incubation and *V* is the reaction velocity.

*t*_1/2_: half-life of enzyme.

ΔH* (kJ mol^−1^) = *E*_*a*_ (45.2 kJ mol^−1^) – *RT*, where *E*_*a*_ is activation energy.

ΔG* (kJ mol^−1^) = − *RT* ln (*k*_*d*_·*h*)/(*k*_*B*_·*T*)

ΔS* is entropy of irreversible inactivation and was calculated from ΔS* = ΔH* − ΔG*/*T*.
